# Sesquiterpene lactones-enriched fractions from *Xanthium mongolicum* Kitag alleviate RA by regulating M1 macrophage polarization *via* NF-κB and MAPK signaling pathway

**DOI:** 10.3389/fphar.2023.1104153

**Published:** 2023-01-26

**Authors:** Jing Han, Jingwen Wang, Yicun Wang, Zhiqi Zhu, Siwang Zhang, Bingrong Wu, Mingsong Meng, Jianning Zhao, Dongsheng Wang

**Affiliations:** ^1^ School of Pharmacy, Nanjing University of Chinese Medicine, Nanjing, China; ^2^ Department of TCMs Pharmaceuticals, School of Traditional Chinese Pharmacy, China Pharmaceutical University, Nanjing, China; ^3^ Department of Orthopedics, Jinling Hospital, School of Medicine, Nanjing University, Nanjing, China

**Keywords:** rheumatoid arthritis, *Xanthium mongolicum* kitag, sesquiterpene lactone, macrophage, traditional Chinese medicine

## Abstract

**Introduction:** Rheumatoid arthritis (RA) is a chronic autoimmune disease, characterized by activated M1-like macrophage in the joint. *Xanthium mongolicum* Kitag (*X. mongolicum*) is a traditional medicinal plant that has long been used to treat RA and other immune diseases in China.

**Methods:** Fractions of *X. mongolicum* were separated based on polarity. Anti-RA activity of the fractions were screened by LPS-stimulated RAW264.7 macrophage *in vitro*. The major active compounds were identified by UPLC-MS and quantified by HPLC. The anti-RA effects of the active fraction was evaluated in complete freund’s adjuvant (CFA)-induced arthritis and collagen-induced arthritis (CIA) mouse models *in vivo* and LPS-stimulated macrophage *in vitro*.

**Results:** Sesquiterpene lactones-enriched fraction from *X. mongolicum* (SL-XM) exhibited the strongest anti-RA activity among all components *in vitro*. Five major constituents i.e., Xanthinosin (1), Xanthatin (2), Mogolide D (3), Mogolide E (4), and Mogolide A (5) were identified as major compounds of SL-XM. SL-XM ameliorated symptoms of CFA and CIA induced arthritis mice model. Furthermore, SL-XM treatment inhibited LPS-induced M1 macrophages polarization. In addition, SL-XM inhibited the phosphorylation of NF-κB and MAPK signaling pathways in LPS-induced macrophage and CIA-challenged mice.

**Discussion:** The main anti-RA active fraction of *X. mongolicum* may be the Sesquiterpene lactones, which includes five key compounds. SL-XM may exert its anti-RA effect by suppressing M1 macrophage polarization *via* the NF-κB and MAPK signaling pathway.

## 1 Introduction

Rheumatoid arthritis (RA) is an autoimmune inflammatory disease that affect about 1% of the world’s population and is more common in women than in men (Chen et al., 2019). With the progression of the disease, more than 90% of RA patients will showed some degree of disability within 20 years of onset, which seriously affects their quality of life. The treatment of early RA is particularly important (Emery et al., 2002). To cope with RA, the main treatments are medication, physical therapy, and lifestyle modification. In terms of drug treatment, which generally include NSAIDs, glucocorticoids (GCs) and DMARDs. Which can effectively relieve the symptoms of RA, but chronic and high dose use of these drugs may cause serious side effects (Ramiro et al., 2017; Mazhar et al., 2018). Biological reagents such as tofacitinib (JAK inhibitor) and anti-TNF-α monoclonal antibodies have proven effective, but have significant side effects and high treatment costs (Puxeddu et al., 2016). Therefore, it is urgent to develop safer, more effective and lower-priced new therapeutic agents to treat RA.

*Xanthium mongolicum* Kitag (*X. mongolicum*) is a common traditional Chinese medicine, which belongs to the genus *Xanthium* (Family Compositae) (Han et al., 2007; Han et al., 2009). According to the Compendium of Materia Medica (Ben Cao Gang Mu, pinyin in Chinese) record, *Xanthium* can be used to treat RA. In addition, Chinese Materia Medica (Zhong Hua Ben Cao, pinyin in Chinese) records that *Xanthium* commonly used in the treatment of inflammatory and immune-related diseases, especially RA (Editorial Committee of Zhonghua Bencao National Traditional Chinese Herb Administration, 1999). Chemical studies have revealed more than 170 chemicals ingredients in *Xanthium* species, including sesquiterpenoids, caffeoylquinic acids, lignans, steroids, thiazinodiones, and flavonoids (Fan et al., 2019). However, to the best of our knowledge, the main anti-RA component of X. *mongolicum* remains unclear.

RA is mainly characterized by chronic inflammation of the synovial joints, ultimately damaging the cartilage and bone (Schett, 2019). Adaptive and innate immune cells participate in persistence and expansion of chronic inflammation in the synovial joints (Calabresi et al., 2018). Among this, macrophages have a dramatic effect on the pathophysiological response to RA, which can polarized into M1/M2 phenotype depending on the microenvironment. M1 macrophages induce early inflammatory responses by secreting pro-inflammatory cytokines, including TNF-a, IL-1β, and IL-6. In contrast, M2 macrophages produce anti-inflammatory cytokines, such as IL-4, IL-10, and IL-13, which are associated with inflammation resolution. Studies have shown that the macrophage in RA joints are predominantly M1 phenotype, which promote RA progression by releasing multiple inflammatory factors. Therefore, targeting macrophages in RA joints has become a promising strategy for its treatment.

In this study, different fractions of the *X. mongolicum* extractions were separated through polarity; Fraction enriched with sesquiterpene lactones from *X. mongolicum* (SL-XM) exhibited the strongest anti-RA activity through *in vivo* experiment. Furthermore, the anti-RA activity of SL-XM was evaluated in CFA and CIA mouse models *in vivo*. Moreover, the possible anti-RA mechanisms of SL-XM was also explored. These results provide experimental evidence for the development of novel anti-RA drugs.

## 2 Materials and methods

### 2.1 Chemicals and reagents

Acetonitrile (34998) and formic acid (00940) for LC-MS were obtained from Merck (WGK, Germany). Incomplete Freund’s adjuvant (IFA), Complete Freund’s adjuvant (CFA), and bovine type II collagen were obtained from Chondrex, Inc. (Redmond, Wash.). ELISA kits for detections of IL-1β (10 × 96t, 88-7013-88), IL-6 (2 × 96t, 88-7064-86), TNF-α (2 × 96t, 88-7324-22), IL-4 (2 × 96t, 88-7044-22) and IL-10 (2 × 96t, 88-7105-22) were purchased from Invitrogen (Karlsruhe, Germany). Anti-IKKα (ab32041, 1:1000), Anti-IKKβ (ab124957, 1/1000), anti-IKKα/β (ab194528, 1:1000), anti-NF-κB p65 antibody (ab16502, 1:1000), anti-P-NF-κB p65 (phospho S536) antibody (ab76302, 1:1000), anti-P38 (ab122517, 1:1000) antibody, anti-P-P38 (phospho Y182) antibody (ab47363, 1:1000), anti-Histone H3 antibody (ab1791, 1:1000) and secondary antibodies were obtained from Abcam (Cambridge, United Kingdom). Anti-IκBα, anti-P-IκBα (phospho S36/32), anti-P-ERK (Thr202/Tyr204), Anti-ERK, anti-JNK, anti- P- JNK (Thr183/Tyr185), anti-STAT6 and anti-P-STAT6 antibodies were obtained from cell signaling technology (Boston, United States). All other reagents and chemicals used were of standard biochemical quality.

### 2.2 General experimental procedures

1D NMR spectra were run on a Bruker DRX-400 spectrometer with TMS as internal standard. LC-MS spectra were run on a Waters Xevo TQD mass spectrometer with Waters ACQUITY UPLC^®^ BEH C18 column and Waters Acquity Arc system equipped with a 2998 PDA detector with Xbridge-C18. Semi-preparative HPLC was performed on an Agilent 1100 with the YMC AA12S05-C18. Column chromatography was performed on silica gel, Sephadex LH-20, or Lichroprep RP-18. Fractions were monitored by thin-layer chromatography (TLC), and spots were detected by spraying with 5% vanillin-sulphuric acid reagent for sesquiterpenes.

### 2.3 Plant material and extraction

**FIGURE 1:**
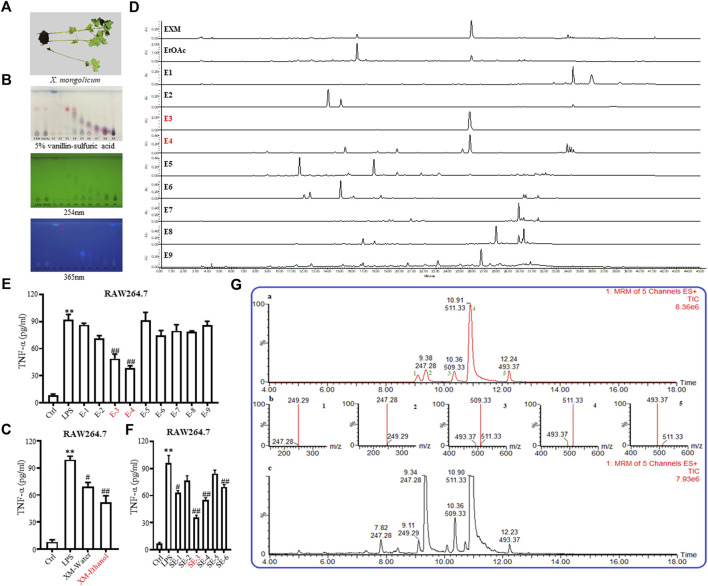
Isolation and identification of SL-XM and the major sesquiterpene lactones. **(A)** Photo of *Xanthium mongolicum* Kitag. **(B,D)** TLC&HPLC analysis showed that sesquiterpene lactones were enriched in E−3 and E4-fractions. ELISA assay of TNF-α in the culture supernatants of RAW264.7 cells incubated with **(C)** Extracts; **(E)** Fractions; **(F)** Sub-fractions of *X. mongolicum* for 4 h and then incubated with LPS(100 ng/mL) for additional 24 h **(G)** LC-MS analysis the main sesquiterpene lactones in *X. mongolicum.*

The aerial parts (stems and leaves without seeds, floral bud and flower) of *X. mongolicum* were harvested at the roadside in Qiqihar (47°41′31.93″ N, 123°93′32.76″ E), Heilongjiang Province, China, in August 2018 (Figure 1A). The specimens were identified by Lingyun Chen, PhD. The voucher specimen (No. JH0002) is stored in Jiangsu Museum of Traditional Chinese Medicine (Nanjing, China).

A total of 10.00 kg of the aerial parts of *X. mongolicum* (without seeds) were collected. After being heated and refluxed, the sample was extracted with 70% ethanol (60 L× 3, for 3, 3, and 2 h) and concentrated by decompression to obtain the crude extract (approximately 0.81 kg). The extract was partitioned successively with ethyl acetate (EtOAc). The EtOAc fraction (0.48 kg) was subjected to silica gel column chromatography eluting with polar petroleum-EtOAc to acquire nine fractions (E1∼E9) (Supplementary Table S3). According to the anti-inflammatory, TLC and HPLC data (Figures 1B, D, E), which showed that fractions E3 and E4 (113.81 g) were enriched in sesquiterpene lactones and that both fractions showed strong anti-inflammatory activity. The fractions E3 and E4 were merged and subjected to silica gel column to obtain six sub-fractions (SE1∼SE6) (Supplementary Table S4). According to the anti-inflammatory data (Figure 1F), the SE3 (16.52 g, SL-XM) was subjected to RP-18 (MeOH-H2O, 10:90-100:0), then to Sephadex LH-20 (MeOH -CHCl_3_, 1:1), further purified by MPLC (YMC XDB-C18, 90 μM, 9.4 mm × 250 mm, 1.8 mL/min, UV detection at 254 and 280 nm) eluting with 50%–60% CH_3_CN, which contained 1.0‰ formic acid to get 1 (1.33 g), 2 (2.79 g), 3 (0.585 g), 4 (0.98 g) and 5 (26.3 mg) (Figure 1G). Compounds were identified by ^1^H NMR (Supplementary Figures S1–5).

Xanthinosin (1): Purity 98.5%; C_15_H_20_O_3_, yellow oil, ^1^H NMR (CDCl_3_, 400 MHz) spectroscopic data see Supplementary Table S1. Positive ESI-MS m/z 249.2 [M + H]^+^ (Shi et al., 2015).

Xanthatin (2): Purity 98.3%; C_15_H_18_O_3_, yellow oil, ^1^H NMR (CDCl_3_, 400 MHz) spectroscopic data see Supplementary Table S1. Positive ESI-MS m/z 247.35 [M + H]^+^ (Yuan et al., 2018).

Mogolide D (3): Purity 97.5%; C_30_H_35_O_7_, white powder, ^1^H NMR (CDCl_3_, 400 MHz) spectroscopic data see Supplementary Table S2. Positive ESI-MS m/z 509.25 [M + H]^+^ (Xu et al., 2017).

Mogolide E (4): Purity 96.4%; C_30_H_37_O_7_, white powder, ^1^H NMR (CDCl_3_, 400 MHz) spectroscopic data see Supplementary Table S2. Positive ESI-MS m/z 509.25 [M + H]^+^ (Xu et al., 2017).

Mogolide A (5): Purity 98.2%; C_30_H_37_O_6_, white powder, ^1^H NMR (CDCl_3_, 400 MHz) spectroscopic data see Supplementary Table S2. Positive ESI-MS m/z 493.25 [M + H]^+^. (Shang et al., 2014).

### 2.4 Analysis SL-XM by UPLC-MS

The identification analysis of subfraction SE3 was carried with a Waters Xevo-TQD MS spectrometer. Each compound was dissolved in the concentration range of 0.01–0.1 μg/mL (in triplicate). The peak areas at 280 nm were plotted against the concentration. The LC conditions: the injection volume of Waters ACQUITY UPLC^®^ BEH C18 column was 2 μL with flow rate of 0.3 mL/min. The mobile phase composed of Pump A (0.1% formic acid in water) and Pump B (acetonitrile). The gradient program started from 10% B, increased to 50% B for 8 min, increased to 100% B for 2 min, kept at 100% B for 2 min, returned to 10% B for 4 min, and kept at 10% B for 2 min. The mass spectrum conditions: Capillary voltage 2.5 kV; nitrogen gas flow rate 800 L/h; dissociation temperature 500 °C; and multiple reaction monitoring ESI positive mode.

### 2.5 Analysis SL-XM by HPLC

The quantitative analysis of the compounds from SL-XM were confirmed by HPLC analysis on the standard compounds, and the ethanolic extract of *X. mongolicum* was dissolved in methanol. The injection volume on Waters X-Bridge C18 column was 10 μL. The wavelengths were detected at 254 and 280 nm. The gradient elution in mobile phase was composed of 0.1% formic acid (solvent A) and acetonitrile (solvent B), with the flow rate set to 0.8 mL/min. The gradient elution program started from 5% B, increased to 50% B for 25 min, increased to 100% B for 5 min, kept at 100% B for 6 min, returned to 5% B for 4 min, and kept under 5% B for 5 min. The results showed that the concentrations of the compounds in SL-XM were 0.133‰ (xanthinosin), 0.279‰ (xanthatin), 0.0585‰ (mogolide D), 0.0981‰ (mogolide E), and 0.00263‰ (mogolide A) respectively as shown in Table 1.

**TABLE 1:** Identification and qualification of sesquiterpene lactone constituents in SL-XM by UPLC.

Peak no Figure 1D	SL-XM component	Mol. formula	Chemical structure	Retention time (min)	Content (‰)
1	Xanthinosin	C_15_H_20_O_3_	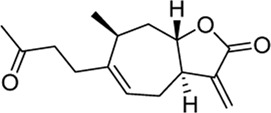	25.409	0.133
2	Xanthatin	C_15_H_18_O_3_	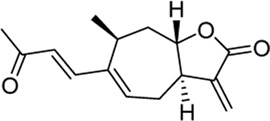	26.012	0.279
3	Mogolide D	C_30_H_36_O_7_	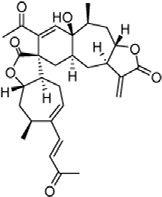	26.841	0.0585
4	Mogolide E	C_30_H_38_O_7_	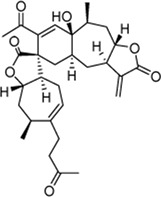	27.603	0.0981
5	Mogolide A	C_30_H_36_O_6_	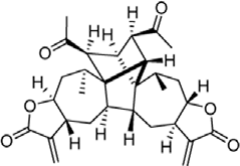	28.133	0.00263

### 2.6 Animals

Male C57/BL6 and DBA/1 mice (8-10 weeks, 20–25 g) were adapted to a 12-hour light-dark cycle in a 23°C ± 2°C environment for a week before use. Mice were raised in a pathogen-free environment with free access to food and water. All experiments were permissioned by the animal ethics committee of Nanjing University of Chinese Medicine (Nanjing, China).

### 2.7 Induction and assessment of CFA

The CFA model was established by subcutaneous injection CFA (Sigma-Aldrich, United States) into the left hind-paw of C57/BL mice as previously reported (Quintao et al., 2019). The control animals received equal amount of saline. Dexamethasone (DXM) is a classic drug for the treatment of RA, so we choose oral administrated DXM as a positive control drug for CFA-challenge mice (Quintao et al., 2019). Han et al. reported effects of *Xanthium strumarium* on acetic acid-induced writhing responses in mice, the doses of chloroform fraction, ethyl acetate fraction, aqueous fraction, and n-Butanol fraction of *Xanthium strumarium* L used were 100, 200, and 400 mg/kg/day in mice (Han et al., 2007). A previous study of ethanolic leaves extract of *X. strumarium* for anti-plasmodial activity used doses of 150, 250, 350 and 500 mg/kg/day in BALB/c mice (Chandel et al., 2012). Muhammad et al. reported effects of methanol extract of the aerial parts of *X. strumarium* on HCl/EtOH-induced mouse model of gastritis, which used doses of 50 and 200 mg/kg/day in mice (Hossen et al., 2016). Based on above studies, we chose 200, 400 mg/kg of SL-XM for mice in our experiments. Mice in SL-XM groups orally received SL-XM (200, 400 mg/kg) daily from day 1 to day 6, and mice in the control and CFA groups received same volume of solvent (*n* = 6 per group). An electronic caliper was used to measure the left hind paw thickness and ankle thickness. Animals were weighed with an electronic scale. In each cases, a trained observer blinded to the experimental groups performed the severity analysis.

### 2.8 Induction and assessment of CIA

CIA model was established in DBA/1 mice as previously reported (Wang et al., 2021). The experimental process is detailed in Figure 6A. Briefly, CFA and bovine type II collagen were fully mixed at a 1:1 ratio. The mice in CIA, SL-XM and DXM groups received an injection of 100 μg emulsified reagent subcutaneously at the root of the tail. 21 days later, the same mice were injected intraperitoneal with 100 μg bovine type II collagen. The control mice received same amount of saline. From day 21 to day 45 after the initial immunization, mice in SL-XM and DXM groups received oral SL-XM (200, 400 mg/kg) or DXM (1 mg/kg) daily. While the control and CIA mice received the same volume of vehicle (*n* = 8 per group). In addition, the arthritis of the mice was evaluated every other day. Paw arthritis was scored 0-4 based on previous report (Atkinson et al., 2012); the scores of the four paws were added to get the mouse arthritis score. The occurrence of arthritis was defined as inflammation of the four paws with an arthritis score of 2 or higher, the incidence of arthritis was the percentage of the diseased mice in all mice. An electronic caliper was used to measure the left hind paw thickness. Animals were weighed with an electronic scale. In each cases, a trained observer blinded to the experimental groups performed the severity analysis.

### 2.9 X rays and Micro-CT

Ankle joint destruction was analyzed by X-ray, radiographs were evaluated on a 0-3 scale as previously reported (Wang et al., 2021). Three-dimensional (3D) image of the mice paws was performed by micro-CT (NFR Polaris-G90).

### 2.10 Histopathological evaluation

The mouse hind paws were harvested at the end of experiment. After immersed in 4% paraformaldehyde for up to 3 days. The tissues were immersed in 17% EDTA at 4°C for 14 days. The paws were paraffin-embedded, cut into 5-μm sections, and stained with H&E using standard protocol. Histopathological images were taken under a Leica DM 4000B photomicroscope. Synovial inflammation, cartilage erosion, and bone erosion were estimated on a 0–3 scale as previously reported (Brenner et al., 2005).

### 2.11 ELISA

Mice blood was taken from the eye socket, and serum was obtained after centrifugation. The concentrations of TNF-α, IL-1β, IL-6, IL-10 and IL-4 were measured with the indicate ELISA kits following the manufacturer’s protocols. After RAW264.7 cells were stimulated with Extracts, Fractions, Sub-fractions of *X. mongolicum* for 4 h and then incubated with LPS for additional 24 h. The culture supernatants were collected for TNF-α ELISA assay kits following the manufacturer’s protocols.

### 2.12 Cell culture

The RAW264.7 macrophages and THP-1 cells were obtained from the Cell bank of the Chinese Academy of Medical Sciences (Shanghai, China). Cells were cultured in RPMI-1640 medium (supplemented with 10% FBS and 1% penicillin-streptomycin) at 37°C in an atmosphere of 95% O_2_ and 5% CO_2_.

### 2.13 Cell viability analysis

RAW264.7 cells were seeded at 10,000 per well in 96-well plates. Plant extract, SL-XM were dissolved in DMSO, and DMSO was used as a control in all experiments at a maximum concentration of 0.1%. After addition of the drug, cells were further incubated for 24 h. Cell viability was detected using the CCK8 kit according to the reagent instructions.

### 2.14 Macrophage polarization toward M1 or M2 phenotype

To explore the effect of SL-XM on the polarization of macrophage towards M1, RAW264.7 or THP-1 cells were stimulated to polarization toward M1 phenotype with LPS (100 ng/mL) for 24 h. At the same time cells were treated with SL-XM. To assess the effect of SL-XM on the polarization of macrophage toward M2, RAW264.7 or THP-1 cells were treated with IL-4 (20 ng/mL) for 24 h, during polarization, macrophages were administrated with SL-XM.

### 2.15 qRT-PCR

Total RNA was extracted from macrophage or ankle tissue using TRIzol reagent, and transcription-PCR was performed by real-time PCR with a standard procedure as previous reported (Wang et al., 2021). Values were normalized to *Gapdh* mRNA levels and calculated by 2^−ΔΔCt^. The primers used are listed in Supplementary Table S3.

### 2.16 Western blot analysis

RAW264.7 cells were planted in a 6-well plate and cultured for 24 h, incubated with SL-XM for 1 h and then added LPS (100 ng/mL) for 30 min, then the cells were harvested. At the end of animal experiment, ankle tissues of different groups were harvested. Proteins were extracted from cells and tissues, and the protein concentration was measured with a BCA kit. Western blot was analysis using the standard protocol as previous reported (Wang et al., 2018).

### 2.17 Immunofluorescence staining

RAW264.7 cells were seeded and cultured on 12 mm glass coverslips, and then cells were fixed with 4% PFA for 10min, permeabilized with Triton-X 100 and blockaded with BSA. After incubated with primary antibody at 4°C overnight, the indicate secondary antibody for additional 2 h, and DAPI for the next 5 min, the samples were transferred onto glass slides and analyzed by fluorescence microscopy analysis.

### 2.18 Flow cytometry

RAW264.7 cells were planted in a 6-well plate and stimulated with IL-4 (20 ng/mL) or LPS (100 ng/mL) for 24h, meanwhile, cells were treated with SL-XM (160 μg/mL). After the cells were prepared into single cell suspension with the density of 10^6^ cells in 100 μL volume in PBS, the cells were incubated with corresponding flow cytometry antibodies on ice for 30 min in dark. After filtration, the stained cells were analyzed with an Attune^®^ NxT Acoustic Focusing Cytometer (Thermo Fisher Scientific, Waltham, MA, United States). The data were further analyzed using FlowJo software version 10.6.2 (Tree Star Inc., Ashland, Or, United States). Antibodies for flow cytometry including BV421-conjugated F4/80, PE-Cy7-conjugated CD86, Alexa 647-conjugated CD206 (San Diego, CA, United States).

### 2.19 Statistical analysis

Statistical analysis was performed using GraphPad Prism 5.01 software. Unless otherwise stated, data are expressed as mean ± standard deviation (SD). Statistical comparative analysis of results between groups were performed using Student’s *t*-test, one-way ANOVA, and two-way ANOVA. Statistically significant was considered when *p* < 0.05.

## 3 Results

### 3.1 Identified the major anti-inflammatory compounds from *X. mongolicum*

To investigate the anti-inflammatory activities of the aerial parts of *X. mongolicum* (Figure 1A), the aerial part of the plant was extracted by water or 70% ethanol. The anti-inflammatory activities of the extract was evaluated by LPS-stimulated macrophages, the result showed the ethanol extract (80 μg/mL) inhibited the secretion of TNF-α in LPS-stimulated macrophages significantly compared with the water extract (80 μg/mL) (Figure 1C). Then, the ethanol extract was isolated by silica gel chromatography to obtain nine fractions (E1-9). TLC and HPLC were applied for analysing sesquiterpene lactones from *X. mongolicum*. After sprayed with 5% vanillin-sulfuric acid reagent, the pink spots on TLC visibly appeared in fraction E3 and E4 (Figure 1B), which showed the main sesquiterpene lactones were enriched in fraction E3 and E4 (Figures 1B, D). At the same time, anti-inflammatory experiments found that fraction E3 and E4 (80 μg/mL) significantly inhibit the secretion of TNF-α in LPS-stimulated macrophages (Figure 1E). Then, fractions E3 and E4 were merged and isolated by silica gel to get six sub-fractions (SE1∼SE6). While the sub-fraction (80 μg/mL) of SE-3 (which depicted as SL-XM later) showed a significant anti-inflammatory activity in LPS-stimulated macrophages (Figure 1F). Then, sub-fraction SE3 was isolated and purified. Five sesquiterpene lactones (Figure 1G) were identified by UPLC-MS as the major constituents. All compounds were isolated using available spectrum data compared with published data. Xanthinosin (1) (Shi et al., 2015), Xanthatin (2) (Yuan et al., 2018), Mogolide D (3) (Xu et al., 2017), Mogolide E (4) (Xu et al., 2017), and Mogolide A (5) (Shang et al., 2014). The compounds 1–5 were quantified by HPLC (Table 1). In addition, the extracts, fractions and sub-fractions of *X. mongolicum* were lyophilized and configured as a solution, and the solution of extracts, fractions and sub-fractions of *X. mongolicum* showed little cytotoxicity at a concentration of 80 μg/mL (data not shown). In addition, RAW264.7 cells were treated with or without different solutions, and the ELSIA results showed the component showed little effect on the production of TNF-α (Supplementary Figures S6).

### 3.2 SL-XM attenuates symptoms of CFA in C57/BL mice

**FIGURE 2:**
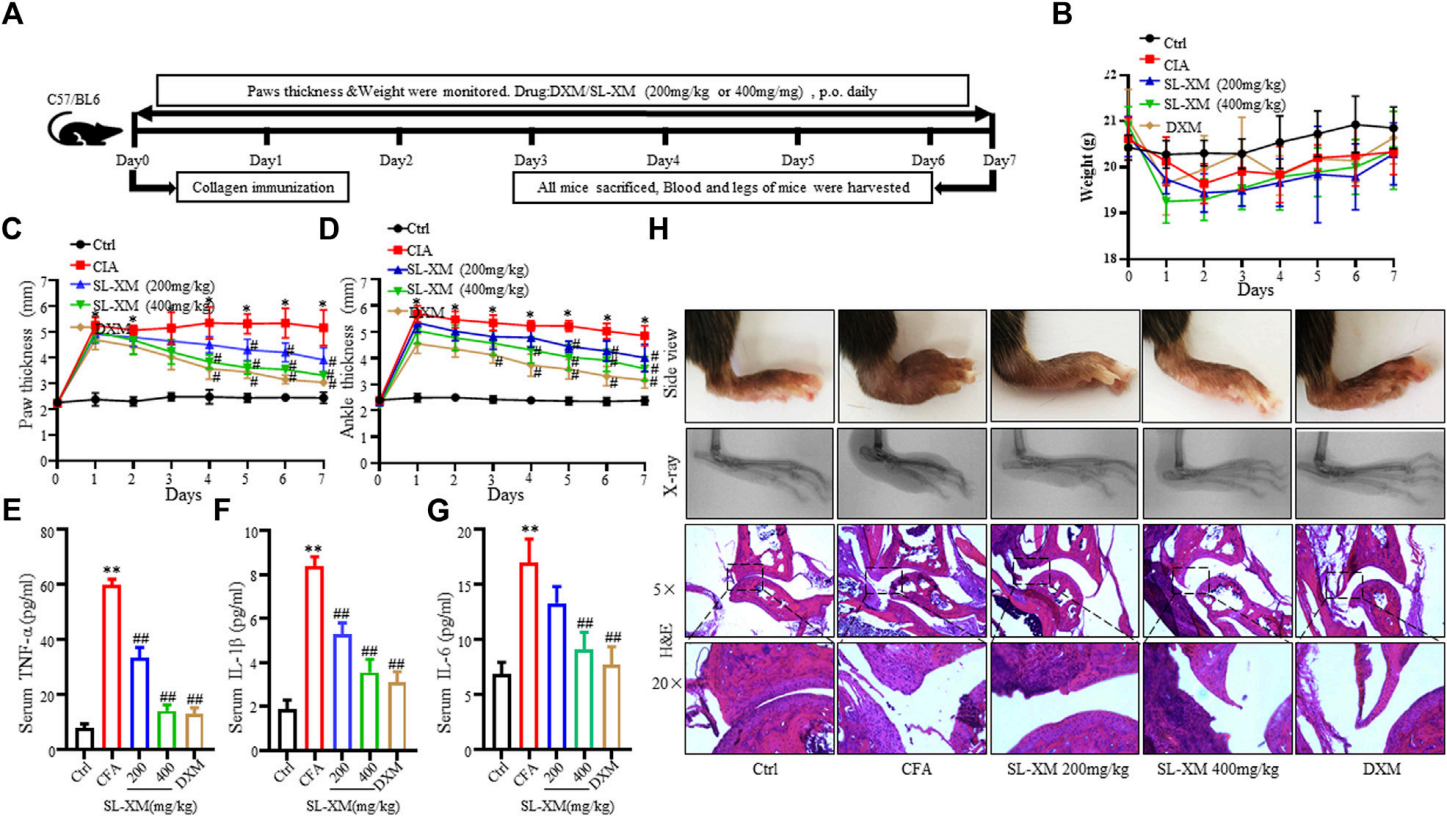
SL-XM dose-dependently attenuates CFA-induced RA in mice. The mice were orally administrated with SL-XM (200, 400 mg/kg) or DXM (1 mg/kg) daily after CFA injection. **(A)** Schematic depiction of the experimental schedule; the body weight **(B)**, Hind paw thickness **(C)**, Ankle thickness **(D)** were measured daily during experiment procedure. Serum levels of TNF-α **(E)**, IL-6 **(F)**, and IL-1β **(G)** were measured 6 days after CFA challenged. **(H)** Representative image of the general features, X-ray and H&E histological graphs of the ankle joint at the end of experiment. *n* = 6 in each group, *p < 0.05, **p < 0.01 vs*.* Ctrl group. ^#^ *p* < 0.05, ^##^ *p* < 0.01 vs*.* CFA group.

CFA-induced arthritis in mice is widely used to screen RA therapeutics. The effect of SL-XM on the symptoms of CFA mice (*n* = 6 per group) was investigated (Figure 2A). The results showed that oral (p.o.) administration of SL-XM (200 and 400 mg/kg) daily reduced paw thickness (Figure 2C); ankle thickness (Figure 2D) and symptom (Figure 2H) in CFA in a dose-dependent manner compared to mice receiving vehicle treatment. X-ray analysis showed that SL-XM attenuates paws bone damage of CFA mice (Figure 2H). H&E analysis showed that SL-XM alleviated paws inflammatory infiltration of CFA mice (Figure 2H). In addition, SL-XM decreased the serum levels of M1 cytokines (IL-1β, IL-6, and TNF-α) in CFA mice (Figures 2E–G). While high-dose SL-XM (400 mg/kg) exhibit similar effect to DXM. During the experiment, there was no significant difference in the weight change of mice in each group (Figure 2B).

### 3.3 SL-XM inhibits M1 macrophage polarization

**FIGURE 3:**
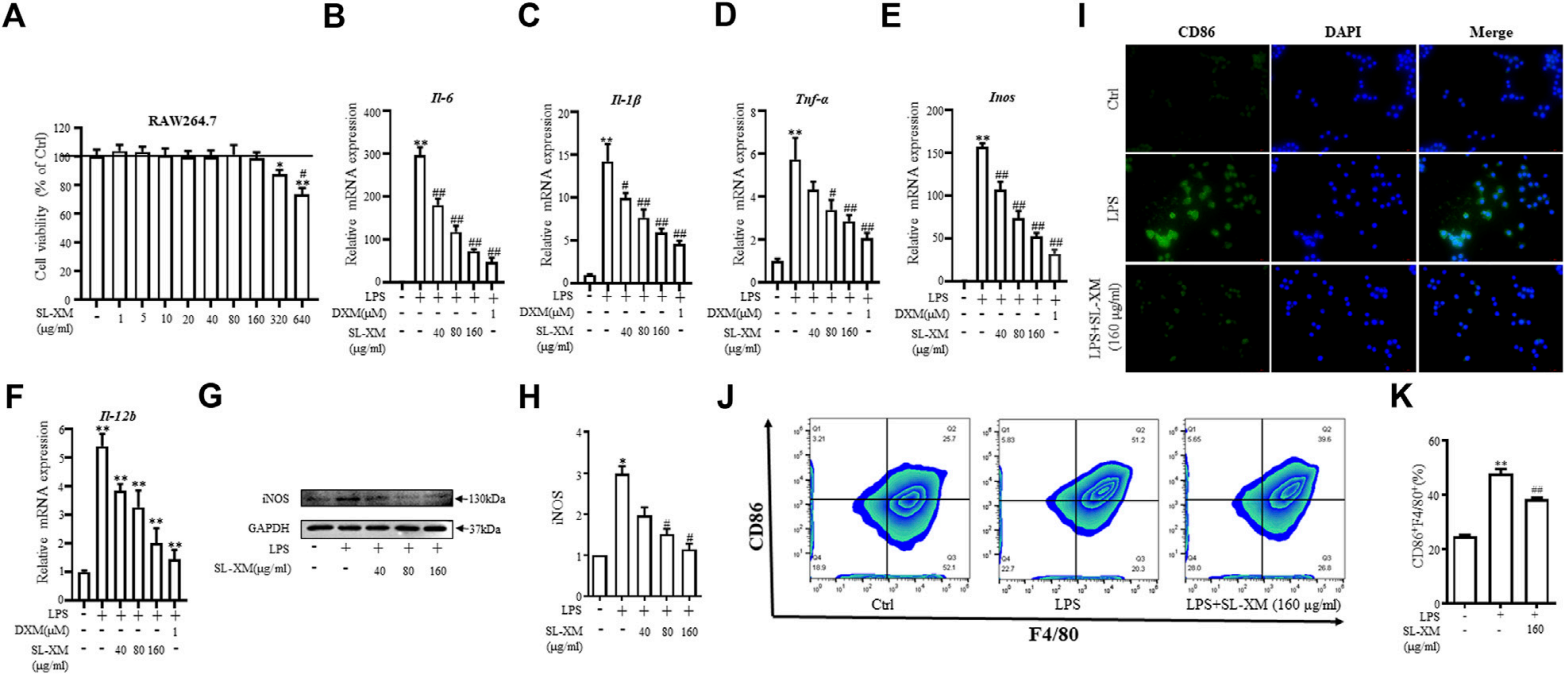
Inhibitory effects of SL-XM on M1 macrophages polarization. **(A)** RAW264.7 cells were stimulated with various concentration of SL-XM for 24 h, and cell viability was measured by CCK8 kit. After RAW264.7 cells were stimulated with LPS (100 ng/mL) and different concentration of SL-XM for 24h, mRNA levels of *Il-6* **(B)**, *Il-1β* **(C)**, *Tnf-α* **(D)**, *Inos* **(E)**, *Il-12b* **(F)** were determined by qRT-PCR; protein level of iNOS **(G,H)** was determined by Western blot analysis; protein level of CD86 **(I)** was determined by immunofluorescence; percentage of CD86-positive cells **(J,K)** was determined by flow cytometry. *p < 0.05, **p < 0.01 vs*.* Ctrl group. ^#^ *p* < 0.05, ^##^ *p* < 0.01 vs*.* LPS group.

To determine the optimal concentration and cytotoxicity of SL-XM, a range of concentrations (1, 5, 10, 20, 40, 80, 160, 320, and 640 μg/mL) were selected for the cytotoxicity tests. As shown in Figure 3A, SL-XM treatments showed no significant toxic effect on macrophage viability below the concentration of 160 μg/mL. qRT-PCR analysis showed SL-XM dose dependently decrease the expression of M1-related genes *Il-6*, *Il-1β*, *Tnf-α*, *Inos* and *Il-12b* in LPS-stimulated RAW264.7 cells (Figures 3B–F). In addition, SL-XM also attenuated the upregulation of *Il-6*, *Il-1β*, *Tnf-α* and *Inos* in LPS-stimulated THP-1 cells (Supplementary Figures S7A–D). Western blot analysis showed that SL-XM dose dependently decreased the LPS stimulated iNOS (Figures 3G, H). Immunofluorescence and flow cytometry demonstrated that SL-XM suppressed CD86 positive cells (M1 macrophages) (Figures 3I–K). These data showed SL-XM inhibited M1 polarization in a dose-dependent manner.

### 3.4 SL-XM promotes M2 macrophage polarization

**FIGURE 4:**
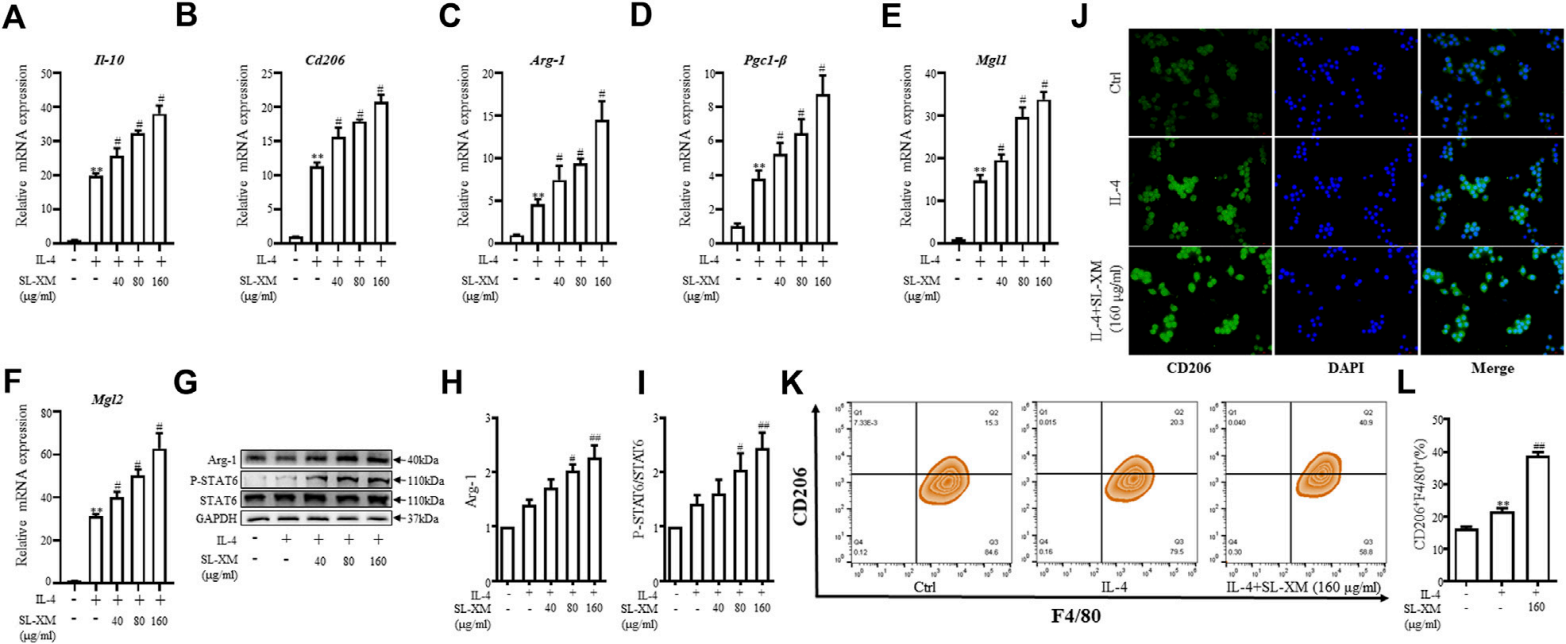
Promotional effect of SL-XM on the polarization of M2 macrophages. After RAW264.7 cells were stimulated with IL-4 (20 ng/mL) and different concentration of SL-XM for 24h, mRNA levels of *Il-10* **(A)**, *Cd206* **(B)**, *Arg-1* **(C)**, *Pgc1-β* **(D)**, *Mgl1* **(E)**, *Mgl2* **(F)** were determined by qRT-PCR; protein level of Arg-1 **(G,H)**, P-STAT6 and STAT6 **(G,I)** was determined by Western blot analysis; protein level of CD206 **(J)** was determined by immunofluorescence; percentage of CD206-positive cells **(K,L)** was determined by flow cytometry. *p < 0.05, **p < 0.01 vs*.* Ctrl group. ^#^ *p* < 0.05, ^##^ *p* < 0.01 vs*.* LPS group.

To explore the effect of SL-XM on the M2 macrophage polarization, macrophage was stimulated with IL-4, followed by treatment with SL-XM. qRT-PCR analysis showed that SL-XM slightly increased the expression of M2-related genes *Il-10*, *Cd206*, *Arg-1*, *Pcg1-β*, *Mgl1* and *Mgl2* (Figures 4A–F). In addition, SL-XM also slightly increased the expression of *Il-10* and *Cd206* in LPS-stimulated THP-1 cells (Supplementary Figures S7A–D). Western blot analysis showed that SL-XM increased the Arg-1 levels in a dose-dependent manner (Figures 4G, H). Immunofluorescence (Figure 4J) and flow cytometry (Figures 4K, L) demonstrated that SL-XM increased CD206 positive cells (M2 macrophages). Subsequently, we examined the molecular expression of M2-related STAT6 pathway (Yang et al., 2017), western blot analysis showed a higher proportion of P-STAT6/STAT6 (Figures 4G, I) in SL-XM-treated macrophages. Which may explain the effect of SL-XM on M2 polarization. These data showed SL-XM slightly increased M2 polarization.

### 3.5 SL-XM negatively regulates LPS-induced M1 macrophage polarization through NF-κB and MAPK signal pathways

**FIGURE 5:**
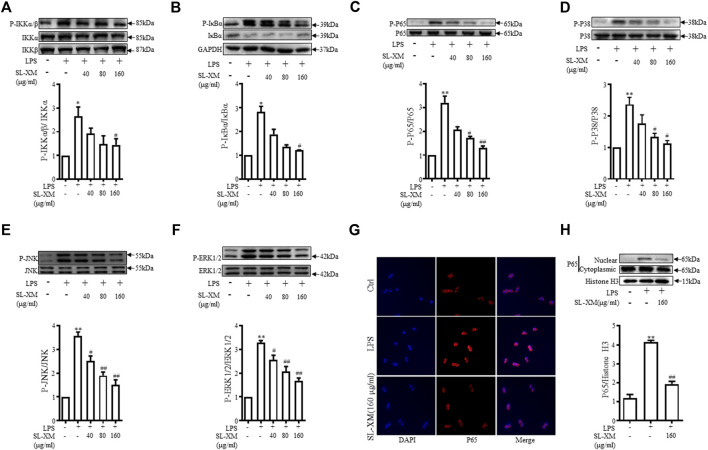
SL-XM regulated LPS-stimulated NF-κB and MAPK signaling pathways in RAW264.7 cells. After RAW264.7 cells incubated with SL-XM for 1 h and then stimulated with LPS (100 ng/mL) for 30 min, protein levels of P-IKKα/β, IKKα, IKKβ **(A)**, P-IκBα, IκBα **(B)**, P-P65, P65 **(C)**, P-P38, P38 **(D)**, P-JNK, JNK **(E)**, P-ERK and ERK **(F)** were determined by Western blot analysis. In addition, NF-κB p65 nuclear translocation was determined by immunofluorescence **(G)** and western blot **(H)**. *p < 0.05, **p < 0.01 vs*.* Ctrl group. ^#^ *p* < 0.05, ^##^ *p* < 0.01 vs*.* LPS group.

NF-κB and MAPKs signaling pathways are classic signaling pathways involved in regulating M1 macrophage polarization. RAW264.7 cells were treated with SL-XM for 1 h, followed by treatment with LPS for 30 min. Western blot analysis showed the phosphorylation of IKKα/β, IκBα, P65, JNK1/2, P38, and ERK in Raw264.7 cells were markedly increased after LPS stimulation. While treated with SL-XM dose dependently inhibited LPS-stimulated phosphorylated IKKα/β, IκBα, P65, JNK, P38, and ERK (Figures 5A–F). In addition, an immunofluorescence assay revealed that SL-XM significantly reduced the nuclear level of P65 in RAW264.7 cells induced by LPS (Figure 5G). We also investigated the abundance of nuclear NF-κB P65 protein by Western blot analysis, the result showed SL-XM significantly reduced the upregulated nuclear level of P65 in RAW264.7 cells induced by LPS (Figure 5H).

### 3.6 SL-XM alleviates symptoms of CIA in DBA/1 mice

**FIGURE 6:**
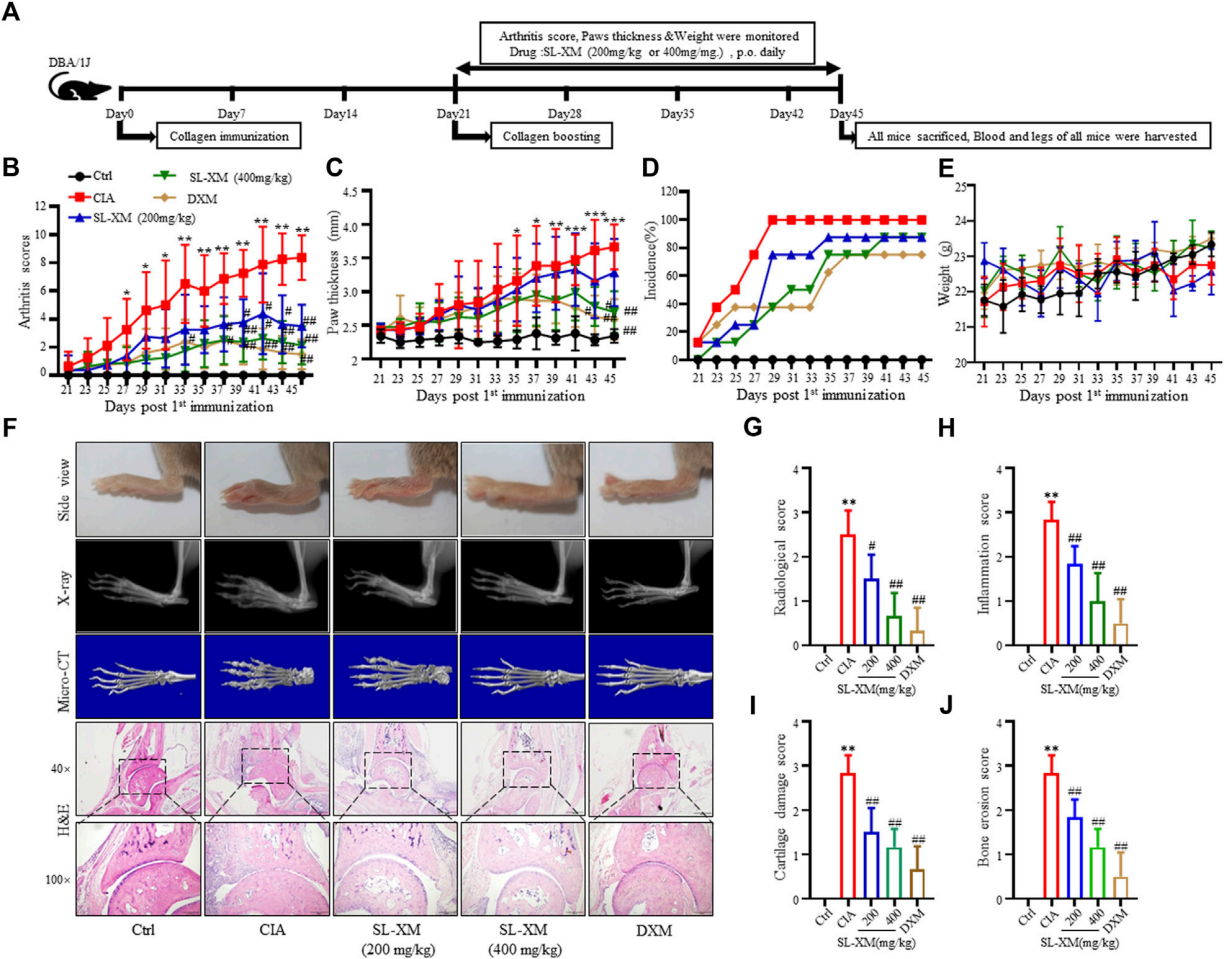
Therapeutic effects of SL-XM on CIA mice. **(A)** Schematic depiction of the experimental schedule. From day 21 to day 45 after the initial immunization **(B)** Arthritis scores, **(C)** Hind paw thickness, **(D)** Onset of CIA symptoms, **(E)** Body weight of mice in each group were evaluated every other day. **(F)** Representative image of the general features, X-ray, micro-CT and H&E histological graphs of the ankle joint at the end of experiment. Mean radiological scores **(G)** of the hind paws of each group. Mean synovial inflammation **(H)**, cartilage erosion **(I)**, and bone erosion scores **(J)** of the hind paws of each group. *n* = 8 in each group, ^*^ *p* < 0.05, ^**^ *p* < 0.01 vs*.* Ctrl group. ^#^ *p* < 0.05, ^##^ *p* < 0.01 vs*.* CIA group.

**FIGURE 7:**
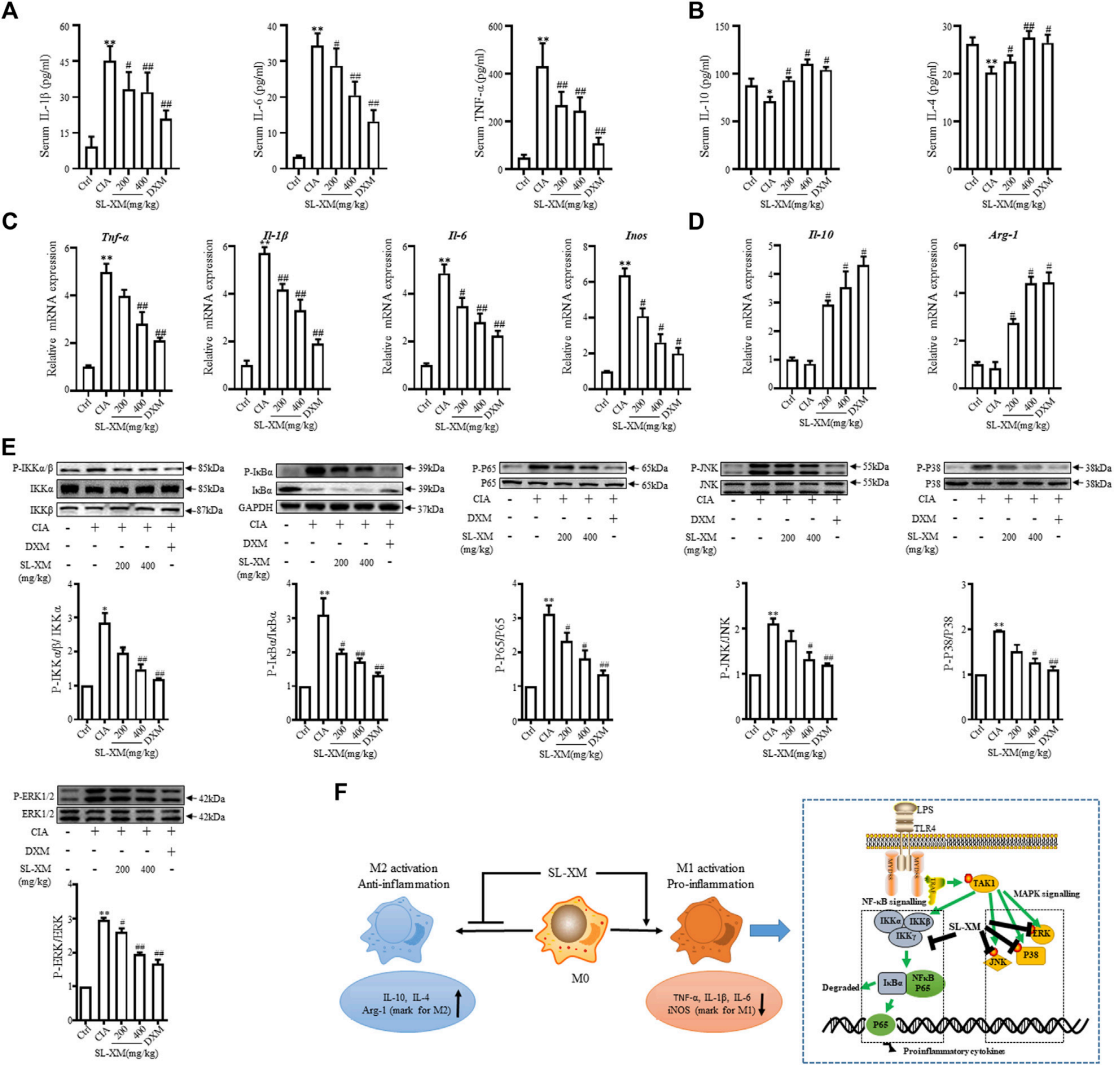
SL-XM reduces M1 cytokines, while increasing M2 cytokines in serum and ankle tissue of CIA mice. At the end of experiment serum level of M1 cytokines (IL-1β, IL-6, and TNF-α) **(A)** and M2 cytokines (IL-10 and IL-4) **(B)** were measured by indicated ELISA kits. At the end of experiment mRNA level of M1 cytokines (*Il-1β*, *Il-6*, *Tnf-α,* and *Inos*) **(C)** and M2 cytokines (*Il-10* and *Arg-1*) **(D)** in the ankle tissue of CIA mice were determined by qRT-PCR. Protein levels of NF-κB and MAPK signaling pathway components in the ankle tissue were determined by Western blot analysis **(E)**. **(F)** Schematic illustration of SL-XM regulates the M1 macrophage polarization through NF-κB to attenuate experimental RA mouse model symptoms. *p < 0.05, **p < 0.01 vs*.* Ctrl group. ^#^ *p* < 0.05, ^##^ *p* < 0.01 vs*.* CIA group.

The CIA mouse model display synovial hyperplasia, cellular infiltration, and cartilage degeneration in the ankle, which are hallmark of RA pathology. The functional role of SL-XM on CIA mice were investigated (Figure 6A). The results showed that p.o. administration of SL-XM (200 and 400 mg/kg) dose-dependently reduced the arthritis score (Figure 6B), paw thickness (Figure 6C), and disease onset (Figure 6D) of CIA mice. In addition, X-ray (Figures 6F, G) and Micro-CT (Figure 6F) analysis showed that oral administration of SL-XM attenuated paws bone damage of CIA mice. H&E staining showed that SL-XM alleviated inflammatory cell infiltration in the paws of CIA mice (Figures 6F, H–J). During the experiment, there was no significant difference in the weight change of mice in each group (Figure 6E). ELISA analysis showed SL-XM treatment attenuated the upregulation of M1 cytokines (IL-1β, IL-6, and TNF-α) (Figure 7A) and the downregulation of M2 cytokines (IL-4 and IL-10) (Figure 7B) in serum. qRT-PCR analysis showed SL-XM treatment reversed the upregulation of M1 cytokines (*Tnf-α*, *Il-1β*, *Il-6* and *Inos*) (Figure 7C) while upregulated M2 cytokine (*Il-10* and *Arg-1*) (Figure 7D) in the ankle tissues. Western blot analysis showed SL-XM treatment attenuated the upregulation of phosphorylation of IKKα/β, IκBα, P65, JNK1/2, P38 and ERK in the ankle tissues of CIA mice (Figure 7E).

## 4 Discussion

In our study, the most potent anti-inflammatory sub-fractions enriched with sesquiterpene lactones was isolated and identified from *X. mongolicum* (SL-XM)*,* and five major sesquiterpene lactones were identified from SL-XM. Furthermore, oral administration of SL-XM significant reduced arthritis symptoms in CFA and CIA model mice. Regarding the mechanism, in RAW264.7 cells and a CIA-induced RA mouse model, SL-XM significantly relieved the upregulated of M1 related cytokines, while slightly restoring the reduction of M2 related cytokines. Suggesting SL-XM relieved RA progression by regulating M1/M2 balance, especially by inhibiting M1 macrophage polarization. In addition, SL-XM blocked the upregulation of phosphorylation of IKKα/β, IκBα, P65, JNK, ERK and P38 in LPS-stimulated RAW264.7 cells and the ankle tissue of CIA mouse model, suggesting SL-XM may exert its anti-RA effect by inhibits M1 macrophage polarization through NF-κB and MAPK signaling pathway.

*Xanthium* is an annual herb that has long been used clinically to treat a range of inflammatory diseases, with many researchers focusing on anti-inflammatory properties. Yeom et al. reported *Xanthii fructus* inhibits inflammatory responses in LPS-stimulated RAW264.7 macrophages through suppressing NF-κB and JNK/p38 MAPK (Yeom et al., 2015). Hossen et al. reported the anti-inflammatory activity of *X. strumarium* methanolic extract (Hossen et al., 2016). *X. mongolicum* is the most widely distributed *Xanthium* species in the north area of China (Han et al., 2009). However, as far as we know, there are few laboratory studies on the phytochemical and pharmacological properties of *X. mongolicum* for its anti-inflammatory, especially anti-RA efficacy. In our study, the ethanol extract of the aerial part of *X. mongolicum* showed stronger anti-inflammatory activity *versus* water extract. Then the ethanol extract was separated to get nine fractions based on the polarity, the active anti-inflammatory fractions were merged and separated to obtain the most active anti-inflammatory sub-fraction (SL-XM). Our study found sesquiterpene lactones were the main anti-inflammatory active ingredient of *X. mongolicum*. This is consistent with previous findings that sesquiterpene lactones were the characteristic and major active metabolites of *Xanthium* species (Merfort, 2011; Schomburg et al., 2013). In terms of the composition of SL-XM, five major sesquiterpene lactones including Xanthinosin, Xanthatin, Mogolide D, Mogolide E and Mogolide A were isolated. As the main compounds in SL-XM, Xanthinosin inhibits iNOS and COX-2 expression and NF-κB activity by inhibiting LPS-induced degradation of IκBα in microglia (Yoon et al., 2008), and Xanthatin alleviated airway inflammation in asthmatic mice by regulating STAT3/NF-κB signaling pathway (Chang et al., 2020), which partially indicates the anti-RA activity of SL-XM.

The imbalance of M1/M2 macrophages in the synovium is related to the inflammation and destruction of RA joints. Previous studies have reported active M1 macrophages express CD86, CD80, and CD64 on their surface and secrete a substantial number of cytokines (such as IL-1β, IL-6, and TNF-α), which promote the onset and progression of RA (Gu et al., 2017). Since LPS is common inducers of M1 macrophage initiation and development, we used LPS to stimulate macrophage to mimic M1 macrophage polarization in the RA. Our study showed that SL-XM significantly reduced M1-related cytokine production, as well as the number of CD86-positive cells in LPS-induced macrophages. Furthermore, M2 macrophages are dominate in promoting tissue remodeling, eliminating inflammatory response and restoring imbalance. As IL-4 plays a key role in the polarization of uncommitted M0 cells into M2 cells (Zhong et al., 2022), it was delighting to find that SL-XM slightly upregulated IL-4 induced M2 macrophage markers, as well as IL-4-induced CD206 positive cells. CFA-induced arthritis is a reliable model to evaluate chronic and acute inflammation leading to joint damage (Quintao et al., 2019), and SL-XM was found dose-dependently alleviate arthritis symptoms in CFA mice. CIA is the most common animal model and its histological features are similar to RA (Miyoshi and Liu, 2018). In the CIA mouse model, SL-XM administered reduced arthritis score and arthritis onset in a dose-dependent manner. In addition, SL-XM treatment reduced immune cell infiltration, cartilage and bone damage in the paw of CIA mice. While high dose of SL-XM showed a similar effect to the treatment of DXM in CIA mice. Moreover, no death or other serious adverse effects were observed in the SL-XM treated mice. These results demonstrate for the first time the significant therapeutic effect of SL-XM on experimental animal model of RA. Macrophage-associated cytokines are primarily produced by macrophages in the inflamed joints and then released into blood, this study found SL-XM strongly inhibited the serum levels of M1-associated cytokines (IL-1β, IL-6 and TNF-α) in CFA and CIA mouse models. We also found SL-XM treatment reversed the upregulation of M1 cytokines (*Tnf-α*, *Il-1β*, *Il-6,* and *Inos*) and the downregulation of M2 cytokine (*Il-10* and *Arg-1*) in the ankle tissues of CIA mouse. These results suggest that SL-XM has a protective effect on RA pathology by regulating macrophage M1/M2 balance, particularly by inhibiting macrophage polarization toward M1.

LPS, as an outer membrane polysaccharide of Gram-negative bacteria, can activate downstream NF-κB and MAPK signaling pathways by binding to the cell surface TLR4 receptor, thereby stimulating cells to produce inflammatory cytokines, chemokines and mediators (Abdollahi-Roodsaz et al., 2007; Yeung et al., 2018). In addition, blocking the NF-κB and MAPK pathways is the key strategy to control the inflammatory response in RA (Fearon et al., 2016). It was found in our study that SL-XM suppressed the LPS-stimulate phosphorylation of IKKα/β, IκBα, P65, P38, ERK and JNK in macrophages *in vitro*. In addition, SL-XM reduced P65 nuclear translocation in LPS stimulated macrophage. These results suggest the protective role of SL-XM in RA may achieved by inhibiting NF-κB and MAPK pathways. Which partially indicate SL-XM inhibits M1 macrophage polarization through NF-κB and MAPK signaling pathway, thereby reducing inflammation and alleviating RA symptoms (Figure 7F).

In our study, we isolated the most potent anti-inflammatory sub-fractions from *X. mongolicum* (SL-XM)*.* The anti-RA effect of SL-XM was evaluated and possible mechanism was explored. For the first time, the anti-RA material base of *X. mongolicum* was explored. We also isolated five major sesquiterpene lactones from SL-XM that have the potential to be developed as anti-RA drug. Nevertheless, more experiments are needed to isolate more active compound from SL-XM, identify key anti-RA compound, and elucidate the anti-RA effects of each compound in the future studies.

Our study offered experimental evidence for the potential therapeutic effects of SL-XM on RA and isolates five major sesquiterpene lactones from SL-XM. The anti-RA effect of SL-XM is related to the regulation of M1/M2 macrophage homeostasis, especially M1 macrophage polarization. In addition, the anti-RA effect of SL-XM by inhibiting M1 macrophage polarization is closely related to the NF-κB and MAPK signaling pathway. These findings showed that SL-XM has a significant therapeutic effect on RA, and provide an experimental basis for the development of new anti-RA drugs from *X. mongolicum.*

## Data Availability

The original contributions presented in the study are included in the article/Supplementary Material, further inquiries can be directed to the corresponding authors.
