# Supplementary material for: Sesquiterpene lactones-enriched fractions from Xanthium mongolicum Kitag alleviate RA by regulating M1 macrophage polarization via NF-κB and MAPK signaling pathway
Source: Front Pharmacol. 2023 Jan 26;14:1104153. doi: 10.3389/fphar.2023.1104153 (PMC9909009; doi:10.3389/fphar.2023.1104153)
Supplement: Supplementary file 1 [file DataSheet1.docx]

Supplementary Material

# Supplementary Figures and Tables

**Supplement-Figure 1** ^1^HNMR of **1**

**Supplement-Figure 2** ^1^HNMR of **2**

**Supplement-Figure 3** ^1^HNMR of **3**

**Supplement-Figure 4** ^1^HNMR of **4**

**Supplement-Figure 5** ^1^HNMR of **5**

**
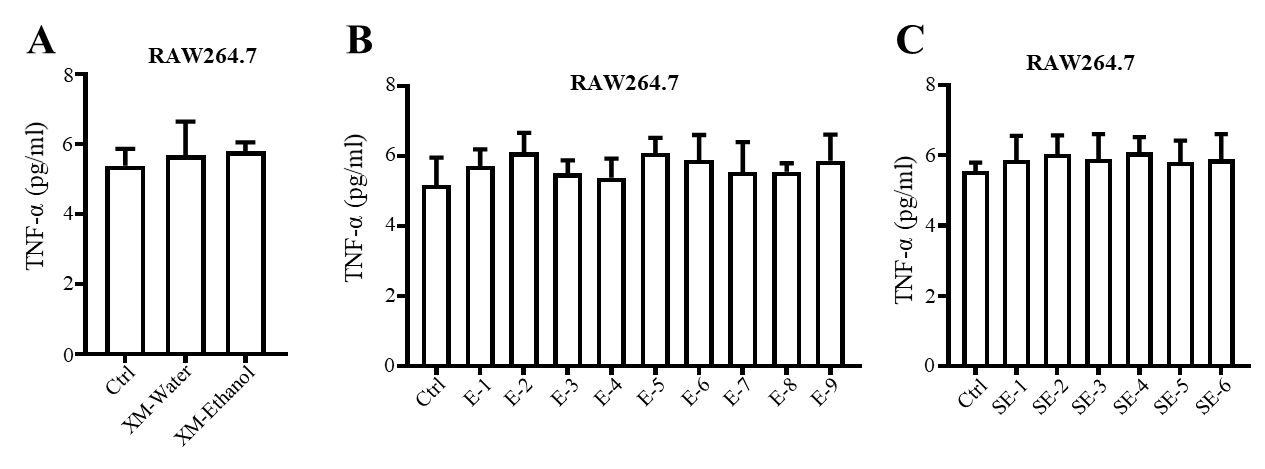
Supplement-Figure 6**.

**Effect of extracts, fractions and sub-fractions of *X. mongolicum* on the TNF-α production of RAW264.7 cells**. ELISA assay of TNF-α in the culture supernatants of RAW264.7 cells incubated with (A) Extracts; (B) Fractions; (C) Sub-fractions of *X. mongolicum* for 24 h.

**
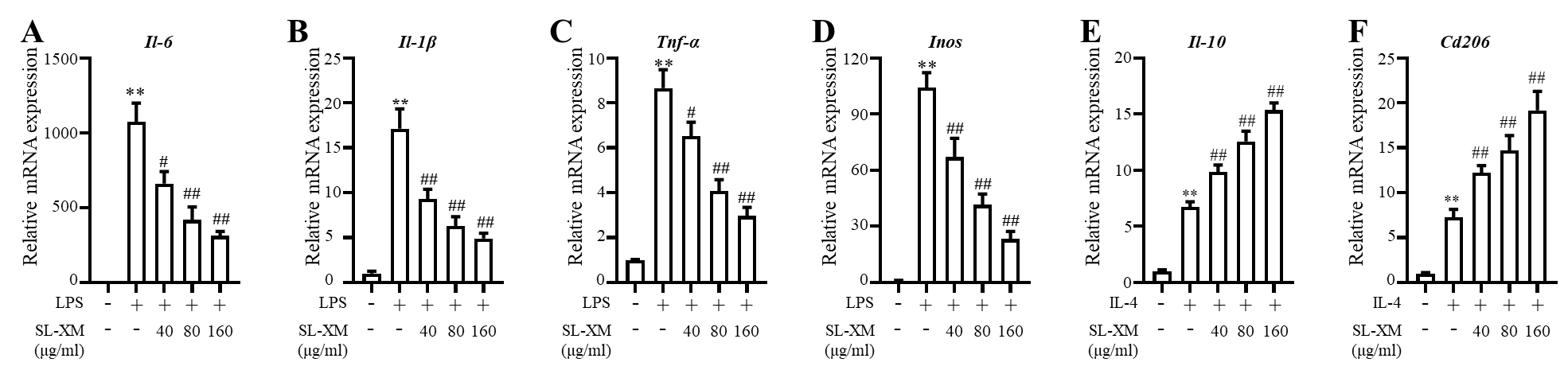
Supplement-Figure 7**.

**Effect of SL-XM on the macrophage polarization toward M1 or M2 phenotype**. After THP-1 cells stimulated with LPS (100ng/mL) and different concentration of SL-XM for 24h, mRNA levels of *Il-6*(A), *Il-1β*(B), *Tnf-α* (C), and *Inos* (D) were determined by qRT-PCR. after THP-1 cells stimulated with IL-4 (20ng/mL) and different concentration of SL-XM for 24h, mRNA levels of *Il-10* (E), *CD206* (F) were determined by qRT-PCR;

**STable 1** ^1^H NMR data of **1**-**2** in chloroform-*d*_1_

| No. | **1** | **2** |
| --- | --- | --- |
|  | *δ*_H_ (*J* in Hz) | *δ*_H_ (*J* in Hz) |
| 1 |  |  |
| 2 | 2.18, m | 7.05, d (16.0) |
| 3 | 2.56, m | 6.19, d (16.0) |
| 4 |  |  |
| 5 | 5.38, d (3.2) | 5.49, d (2.8) |
| 6 | 2.39, m | 2.38, m |
|  | 2.52, m | 2.54, m |
| 7 | 2.67, m | 2.79, ddd (16.8, 9.2, 2.4) |
| 8 | 4.18, ddd (12.8, 10.0, 2.8) | 4.28, ddd (12.4, 10.4, 2.4) |
| 9 | 2.49, m | 1.84, m |
|  | 2.13, m | 2.21, m |
| 10 | 2.29, m | 3.07, m |
| 11 | 1.70, td (12.4, 4.0) | 1.15, d (7.6) |
| 12 |  |  |
| 13 |  |  |
| 14 | 2.10, s | 2.30, s |
| 15 | 5.47, dd (7.6, 3.2)  6.07, d (3.2) | 5.49, d (2.8)  6.29, dd (9.2, 2.8) |

**STable 2** ^1^H NMR data of **3**-**5** in chloroform-*d*_1_

| No. | **3** | **4** | **5** |
| --- | --- | --- | --- |
|  | *δ*_H_ (*J* in Hz) | *δ*_H_ (*J* in Hz) | *δ*_H_ (*J* in Hz) |
| 1 |  |  |  |
| 2 | 6.81, s | 6.76, s | 2.97 (d, 2.4) |
| 3 |  |  | 2.93 (dd, 6.6, 2.4) |
|  |  |  |  |
| 4 |  |  |  |
| 5 | 2.31, m | 2.20, m | 1.99 (m) |
| 6 | 2.34, m | 2.44, m | 2.12 (m) |
|  | 1.28, m | 1.64, m |  |
| 7 | 3.40, t (10.0) | 3.36, t (10.0) | 2.69 (m) |
| 8 | 4.13, brtd (4.0, 10.0, 12.4) | 4.13, ddd (4.0, 10.0, 12.4) | 4.20 (ddd, 11.8, 9.1, 2.4) |
| 9 | 2.49, m | 2.50, m | 2.29 (m) |
|  | 2.13, m | 2.07, m | 1.85 (m) |
| 10 | 2.29, m | 2.26, m | 2.52 (m) |
| 11 |  |  |  |
| 12 |  |  |  |
| 13 | 6.16, d (3.2) | 6.18,d (3.2) | 6.22 (d, 3.1) |
|  | 5.46, d (3.2) | 5.43, d (3.2) | 5.48 (d, 3.1) |
| 14 | 1.12, d (7.6) | 1.13, d (7.2) | 1.06 (d, 7.0) |
| 15 | 2.37, s | 2.37, s | 1.97 (s) |
| 1‘ |  |  |  |
| 2’ | 7.00, d (15.6) | 2.18, m 1.97, m | 3.14 (d, 6.6) |
| 3’ | 6.21, d (15.6) | 2.49, m 2.41, m | 2.83 (s) |
| 4’ |  |  |  |
| 5’ | 6.13, dd (2.4, 9.2) | 5.36, dd (2.0, 9.2) | 2.29 (m) |
| 6’ | 2.18, brt (14.8) | 2.28, m | 1.98 (m) |
|  | 2.59,dd (10.0, 14.8) | 1.28, m | 1.80 (m) |
| 7’ | 2.40, m | 2.43, m | 2.69 (m) |
| 8’ | 4.53, brtd (1.6, 10.8) | 4.48, td (2.8, 10.0) | 4.08 (m) |
| 9’ | 1.85, td (2.8, 10.8) | 1.79, m | 2.28 (m) |
|  | 2.38, m | 2.36, m |  |
| 10’ | 3.02, m | 2.25, m | 2.85 (m) |
| 11’ |  |  |  |
| 12’ |  |  |  |
| 13’ | 1.68, t (14.0) | 2.35, m | 6.16 (d, 3.2 Hz) |
|  | 2.05, d (14.0) | 2.25, m | 5.37 (d, 3.2 Hz) |
| 14’ | 1.16, d (7.6) | 1.14, d (7.2) | 0.95 (d, 7.3) |
| 15‘ | 2.28, s | 2.15, s | 2.32 (s) |

**STable 3** The solvent and fractions isolated from XM

| **Fractions** | **Ratio of petroleum-EtOAc** | **Weight/g** |
| --- | --- | --- |
| E1 | 1: 0 | 70.30 |
| E2 | 30:1, 20:1,10:1 | 35.90 |
| E3 | 5:1 | 37.94 |
| E4 | 5:1 | 75.87 |
| E5 | 3:1 | 34.40 |
| E6 | 3:1 | 44.56 |
| E7 | 1:1 | 40.63 |
| E8 | 1:1 | 26.82 |
| E9 | 0:1 | 94.22 |

**STable 4** The solvent and sub-fractions isolated from E3

| **Sub-Fractions** | **Ratio of petroleum-EtOAc** | **Weight/g** |
| --- | --- | --- |
| SE1 | 10: 1 | 8.83 |
| SE2 | 5:1 | 15.47 |
| SE3 | 5:1 | 16.52 |
| SE4 | 3:1 | 9.25 |
| SE5 | 3:1 | 28.55 |
| SE6 | 1:1 | 32.60 |

**STable 5** The primers of genes used in the article

| Gene | Forward primer (5’ to 3’) | Reverse primer (5’ to 3’) |
| --- | --- | --- |
| Mouse *Gapdh* | GGGTCCCAGCTTAGGTTCAT | CCAATACGGCCAAATCCGTT |
| Mouse *Il-1β* | ATGAAGGGCTGCTTCCAAAC | TCTCCACAGCCACAATGAGT |
| Mouse *Il-6* | GGAGCCCACCAAGAACGATA | ACCAGCATCAGTCCCAAGAA |
| Mouse *Tnf-α* | CTCATGCACCACCATCAAGG | ACCTGACCACTCTCCCTTTG |
| Mouse *Inos* | GCGCTCTAGTGAAGCAAAGC | TGCAACAGCTGAGGAAGGACTTGA |
| Mouse *Il-12b* | GTGGAATGGCGTCTCTGTCT | GGTCTGGTTTGATGATGTCCCT |
| Mouse *Arg-1* | TGGCTTGCGAGACGTAGAC | GCTCAGGTGAATCGGCCTTT |
| Mouse *Il-10* | CTGGACAACATACTGCTAACCG | GGGCATCACTTCTACCAGGTAA |
| Mouse *Cd206* | CAGCGGTTGGCAGTGGA | CAGCTGATGGACTTCCTGGTAAG |
| Mouse *Arg-I* | AGGCGCTGTCATCGATTTCT | TGGAGTCCAGCAGACTCAAT |
| Mouse *Pgc1β* | CTTCCGTTGGCCCAGATAC | CTGCTGGGCCTCTTTCAGTA |
| Mouse *Mgl1* | TGCAACAGCTGAGGAAGGACTTGA | AACCAATAGCAGCTGCCTTCATGC |
| Mouse *Mgl2* | GCATGAAGGCAGCTGCTATTGGTT | TAGGCCCATCCAGCTAAGCACATT |
| Human *iNOS* | AGCTGAACTTGAGCGAGGAG | GGAAAAGACTGCACCGAAGA |
| Human *Tnf-α* | CTCTTCTGCCTGCTGCACTTTG | ATGGGCTACAGGCTTGTCACTC |
| Human *Il-1β* | ATGATGGCTTATTACAGTGGCAA | GTCGGAGATTCGTAGCTGGA |
| Human *Il-6* | ACTCACCTCTTCAGAACGAATTG | CCATCTTTGGAAGGTTCAGGTTG |
| Human *Cd206* | GGCGG TGACC TCACA AGTATCC | TCATG GCTTG GTTCT CCACG AA |
| Human *Il-10* | TCTCCGAGATGCCTTCAGCAGA | TCAGACAAGGCTTGGCAACCCA |
| Human *Gapdh* | AACTCCCACTCTTCCACCTTCG | TCCACCACCCTGTTGCTGTAG |
